# Dynamic C-reactive protein trajectories predict prolonged healing time in diabetic wounds: a machine learning model based on a single-center cohort with standardized wound size

**DOI:** 10.3389/fmed.2026.1778003

**Published:** 2026-02-12

**Authors:** Sichao Jiang, Qixuan Song, Junhuan Wang, Xiaohong Sun, Zhen Zhang, Shouyu Wang, Junwei Zong

**Affiliations:** 1 Department of Orthopaedic Surgery, The First Affiliated Hospital, Dalian Medical University, Dalian, China; 2 Department of Obstetrical, Affiliated Hospital, Qingdao University, Qingdao, China

**Keywords:** cohort analysis, CRP dynamics, diabetes mellitus, machine learning model, wound prognosis

## Abstract

**Objective:**

To develop a machine learning (ML) model for predicting prolonged healing (>8 weeks) in diabetic wounds, focusing on dynamic C-reactive protein (CRP) trajectories.

**Methods:**

This was a retrospective single-center cohort study. We included 465 patients with type 2 diabetes, standardized wound sizes (5–8 cm^2^), and debridement alone (2021–2024: training set, *n* = 325; 2025: temporal validation set, *n* = 140). Serial CRP was measured at admission (CRP), post-antibiotic preoperatively (CRP_2nd), and postoperatively at discharge (CRP_3rd). Therapeutic response variables (therapeutic_response_1/2/all) were calculated as percentage changes in serial CRP levels across treatment phases, reflecting anti-inflammatory/antimicrobial efficacy. LASSO regression selected features, 12 ML models were constructed, and performance was evaluated via AUC, sensitivity, and specificity. SHAP analysis interpreted predictions.

**Results:**

The GradientBoosting model exhibited superior performance (validation set: accuracy = 0.9357, sensitivity = 0.8689, specificity = 0.9873). LASSO regression identified 15 key variables [including CRP_2nd, CRP_3rd, albumin (ALB)]. SHAP analysis revealed CRP_2nd as the most influential predictor (mean absolute SHAP value = 0.460), with elevated CRP_2nd/CRP_3rd associated with prolonged healing and higher ALB/favorable therapeutic responses as protective factors.

**Conclusion:**

Dynamic CRP trajectories, particularly CRP_2nd, are critical for predicting prolonged diabetic wound healing. The GradientBoosting model provides a clinically actionable tool for risk stratification.

## 1 Introduction

C-reactive protein (CRP), produced by hepatocytes in response to proinflammatory cytokines like interleukin-6 (IL-6) and tumor necrosis factor-α (TNF-α), is a well-established biomarker of systemic inflammation. Its rapid elevation during infection, tissue injury, or inflammation, followed by a decline with effective treatment, makes it a cornerstone of clinical monitoring for disease activity and therapeutic response (1). In the context of wound healing, CRP dynamics reflect the balance between inflammatory clearance and tissue repair—an equilibrium particularly fragile in chronic wounds, where persistent inflammation often impedes progression to the proliferative and remodeling phases (2).

Diabetic wounds, such as foot and lower extremity skin ulcers, are a significant complication of diabetes mellitus, impacting up to 25% of patients during their lifetime (3). These wounds exhibit impaired angiogenesis, neuropathy, and dysregulated inflammation, resulting in extended healing durations, frequent recurrences, and an elevated risk of amputation (4). The clinical burden is substantial: even small-to-moderate diabetic wounds (5–8 cm^2^) can require weeks to months to heal, with approximately 30%–40% of cases progressing to prolonged healing (>8 weeks) despite standard care (5, 6). This unpredictability not only increases healthcare costs but also diminishes patients’ quality of life, underscoring the need for reliable tools to identify high-risk individuals early.

Existing research on diabetic wound healing has primarily focused on static baseline factors, such as initial wound size, depth, and comorbidities like peripheral artery disease (7). While these factors provide valuable context, they fail to capture the dynamic biological processes that drive healing outcomes. In recent years, studies have begun to explore inflammatory markers as predictors: for example, elevated baseline CRP levels have been associated with prolonged healing (8), and serial measurements of proinflammatory cytokines (e.g., IL-6, TNF-α) have shown promise in assessing treatment response (9). However, these studies often lack standardization in wound size, include heterogeneous patient populations (e.g., mixing minor debridement with major amputations), or rely on single-time-point measurements, limiting their clinical applicability.

The importance of dynamic inflammatory trajectories—rather than isolated values—has gained recognition in other chronic conditions, such as sepsis and rheumatoid arthritis, where patterns of marker decline (e.g., CRP “clearance rate”) strongly predict outcomes (10, 11). In diabetic wounds, this principle is equally relevant: an initial elevation in CRP may indicate a productive inflammatory response to clear pathogens, but a failure to decline after interventions (e.g., antibiotics, debridement) signals persistent infection or impaired healing mechanisms (12). Surprisingly, few studies have systematically analyzed serial CRP measurements to predict prolonged healing, especially in well-characterized cohorts with standardized wound sizes and uniform surgical interventions (e.g., debridement alone, excluding amputations).

Machine learning (ML) has become a valuable asset in wound healing research, surpassing traditional regression models by effectively capturing nonlinear relationships and interactions among multiple variables. Existing ML models for diabetic wounds have integrated features such as wound imagery, microbiome data, and baseline clinical metrics to predict healing time or amputation risk (13–15). For instance, a recent study using random forest algorithms achieved an AUC of 0.82 for predicting 12-week healing in diabetic foot ulcers, leveraging features like wound area and HbA1c (13). However, these models rarely incorporate dynamic inflammatory markers, and none have specifically focused on CRP trajectories across critical treatment time points (e.g., post-antibiotic, pre-operative, and post-operative phases).

Our study seeks to create a machine learning model to predict diabetic wound healing times exceeding 8 weeks, emphasizing the dynamic patterns of CRP. By restricting the cohort to patients with standardized wound sizes (5–8 cm^2^) who underwent only debridement (excluding amputations), we minimize confounding by wound severity and surgical complexity. We hypothesize that serial CRP measurements—specifically the magnitude and rate of decline after antibiotic treatment and surgery—will outperform static baseline factors in predicting prolonged healing. This model will not only enhance risk stratification but also highlight critical intervention windows, enabling clinicians to adjust anti-inflammatory and antimicrobial strategies proactively. Our objective is to develop a clinically useful tool to enhance outcomes for patients with diabetic wounds. While we utilized a temporal validation cohort to assess model robustness, the single-center design warrants future multi-center replication to confirm generalizability across diverse clinical settings.

## 2 Materials and methods

### 2.1 Data source

This retrospective cohort study utilized clinical data from patients at the First Affiliated Hospital of Dalian Medical University. The training dataset comprised patients hospitalized from January 2021 to December 2024, and a temporal validation dataset included patients admitted in 2025 to evaluate the model’s generalizability within the same institutional setting. Notably, this constitutes temporal validation (time-separated, single-center) rather than multi-center external validation, which will be addressed in future research. This retrospective cohort study was conducted in accordance with the Helsinki Declaration of 1975, as revised in 2024. The study protocol was approved by the Ethics Committee of the First Affiliated Hospital of Dalian Medical University (Approval No.: PJ-KS-KY-2025-440). Due to the retrospective nature and use of de-identified data, the requirement for informed consent was waived by the Ethics Committee, with strict adherence to patient anonymity and data protection principles.

### 2.2 Participants

**Figure 1:**
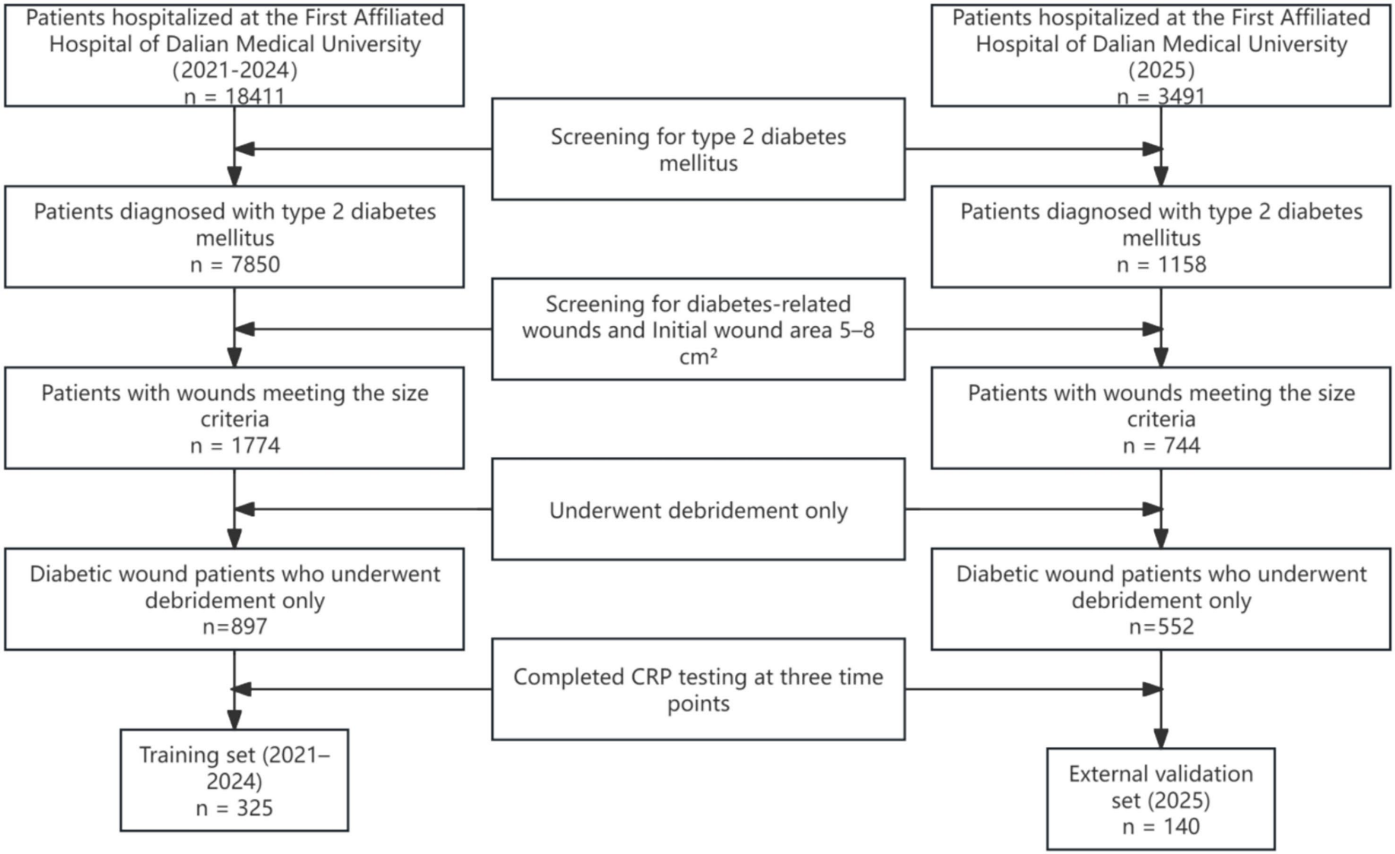
Patient selection flowchart for retrospective cohort study on diabetic wound healing.

The patient selection process is illustrated in Figure 1.

#### 2.2.1 Inclusion criteria

Met WHO criteria for type 2 diabetes mellitus, defined by fasting blood glucose ≥7.0 mmol/L or HbA1c ≥ 6.5%, excluding type 1, gestational, or secondary diabetes.

Admitted for diabetes-related wounds (e.g., diabetic foot ulcers, lower extremity skin ulcers) with an initial wound area of 5–8 cm^2^, Wound area was measured by two experienced attending physicians independently using ImageJ software (version 1.8.0), both of which were uniformly trained before measurement, using the same image acquisition and measurement criteria (e.g., the area not covered by epithelization of the wound edge was used as the measurement range). The intraclass correlation coefficient (ICC) was used to evaluate the measurement consistency, and the ICC was 0.96 (95% CI: 0.94–0.98), indicating that the measurement results of the two reviewers were highly consistent;

Underwent debridement only (excluding major surgical interventions such as amputation, digit resection, or skin flap transplantation);

Completed C-reactive protein (CRP) testing at three time points during hospitalization:

① At admission (within 24 h of admission, denoted as CRP);
② After antibiotic treatment but before surgery (typically 3–7 days after admission, denoted as CRP_2nd);
③ At discharge after surgical treatment (wound not fully healed but meeting discharge criteria, denoted as CRP_3rd);

CRP_2nd was measured from 3 to 7 days after admission (median 4 days, interquartile range: 3 to 5 days) and was strictly recorded and included in the database for all patients. One-way analysis of variance was used to test the correlation between the measurement time (3, 4, 5, 6–7 days) and CRP_2nd values. The results showed that there was no statistically significant difference in CRP_2nd levels at different time points (*F* = 1.23, *p* = 0.301). This indicates that measurement variability within this time window did not significantly influence the clinical significance of CRP_2nd.

Aged ≥18 years with complete clinical data and at least 12 weeks of follow-up to record the final healing time.

#### 2.2.2 Exclusion criteria

Complicated with malignant tumors, severe immunodeficiency diseases (e.g., AIDS), or end-stage liver/kidney failure (factors independently affecting wound healing);

Death or transfer to another hospital due to non-wound-related causes during treatment (inability to obtain complete healing time);

Abandonment of treatment or loss to follow-up before wound healing;

Presence of other acute diseases affecting inflammatory markers (e.g., acute myocardial infarction, pneumonia).

### 2.3 Variable selection and definition of target variables

#### 2.3.1 Collected variables

Variables were selected based on three principles: (1) Clinical relevance: indicators known to be associated with diabetic wound healing (e.g., inflammatory markers, nutritional markers, metabolic markers, etc.) were included; (2) Data availability: all included variables were clinical routine indicators to ensure the clinical practicability of the model. (3) Literature support: refer to the variable selection experience of previous diabetic wound prediction models. Indicators such as wound culture results were not included because some patients lacked complete culture data (missing rate > 30%), which may introduce bias. Rare comorbidities, such as rare autoimmune diseases, were not included, which had limited contribution to model prediction because their incidence was less than 1%.

Clinical data were obtained from electronic medical records, encompassing;

Demographic information: gender, age, smoking history, drinking history;

Biochemical indicators: The study examines various biomarkers, including low-density lipoprotein cholesterol (LDL-C), triglycerides (TG), albumin (ALB), albumin/globulin ratio (ALB/GLO), direct bilirubin (DBIL), alkaline phosphatase (ALP), glucose (Glu), high-sensitivity cardiac troponin I (hs-cTnI), procalcitonin (PCT), high-density lipoprotein cholesterol (HDL-C), D-dimer, and several hematological parameters such as mean corpuscular volume (MCV), mean corpuscular hemoglobin (MCH), mean corpuscular hemoglobin concentration (MCHC), mean platelet volume (MPV), red blood cell distribution width (RDW-CV and RDW-SD), hematocrit (Hct), red blood cell count (RBC), platelet count (PLT), hemoglobin (Hb), and electrolytes including chloride (Cl^−^), sodium (Na^+^), potassium (K^+^), magnesium (Mg^2+^), and glycated hemoglobin (HbA1c).

Hematological indicators include neutrophil percentage (Neut%), neutrophil count (Neut#), monocyte percentage (Mono%), monocyte count (Mono#), basophil percentage (Baso%), basophil count (Baso#), eosinophil percentage (Eos%), eosinophil count (Eos#), lymphocyte percentage (Lymph%), lymphocyte count (Lymph#), white blood cell count (WBC), erythrocyte sedimentation rate (ESR), and globulin (GLO).

Inflammatory dynamic indicators: CRP (measured at admission within 24 h), CRP_2nd (measured 3–7 days after admission, post-antibiotic preoperatively), CRP_3rd (measured at discharge postoperatively), therapeutic response at first stage (therapeutic_response_1), therapeutic response at second stage (therapeutic_response_2), overall therapeutic response (therapeutic_response_all). All CRP measurements were performed using a standardized nephelometric assay (Siemens BNII System) with consistent reagents and quality control protocols throughout the study period (2021–2025). The inter-assay coefficient of variation (CV) was < 5%, and the lower limit of detection was 0.1 mg/L, ensuring reliability and comparability of serial measurements.

Data preprocessing flow: (1) Missing value handling: Variables with a missing rate <5% were imputed using multiple imputation by chained equations (MICE) to preserve data integrity; variables with a missing rate >5% were excluded to avoid bias from excessive imputation. This 5% threshold was selected based on established practices in clinical prediction model research and validated via sensitivity analysis: imputing variables with missing rates > 5% increased training set accuracy to 0.981 but reduced validation set accuracy to 0.887 (vs. 0.9357 for the final model), indicating heightened overfitting risk; (2) Outlier handling: Identify outliers using the IQR method and handle them through Winsorization (scaling to the 99th percentile) to avoid interference from extreme values; (3) Feature standardization: Perform *Z*-score standardization on continuous variables to eliminate dimensional differences; (4) Categorical variable encoding: Use one-hot encoding to handle binary categorical variables such as gender and smoking history.

#### 2.3.2 Definition of therapeutic response variables

Therapeutic response variables were rule-based metrics derived from serial CRP changes to quantify treatment efficacy across critical phases: Therapeutic_response_1: Percentage change in CRP from admission (CRP) to post-antibiotic preoperatively (CRP_2nd). A positive value indicates a decrease in CRP (favorable response), while a negative value indicates an increase (unfavorable response). Therapeutic_response_2: Percentage change in CRP from post-antibiotic preoperatively (CRP_2nd) to discharge (CRP_3rd). Therapeutic_response_all: Composite therapeutic response, defined as the weighted average of therapeutic_response_1 and therapeutic_response_2, with weights proportional to the duration of the antibiotic treatment phase (3–7 days) and postoperative recovery phase (until discharge).

#### 2.3.3 Definition of target variable

The primary outcome was “prolonged healing,” defined as failure to achieve complete wound healing (full epithelialization without exudate) within 8 weeks (56 days) after initial debridement. Healing status was confirmed through 12-week follow-up (in-person or telephone interviews).

### 2.4 Variable screening using LASSO regression

LASSO regression, a regularized linear regression approach that is extensively utilized for variable selection, conducts variable selection and complexity adjustment during the fitting of generalized linear models. In this research, LASSO regression was employed for variable screening. The optimal penalty parameter λ of LASSO regression was determined by 10-fold cross-validation. The classification error was used as the objective function, and the value of λ corresponding to the minimum error (λ = 0.012) was selected. The formula for LASSO regression is presented below:


$$\min_{\beta }\{\frac{1}{2n}y+X\beta_{2}^{2}+\lambda \beta_{1}\}$$

In the equation, y denotes the response variable vector indicating whether healing is prolonged. X is the design matrix with observations of p explanatory variables for n samples. *β* is the regression coefficient vector, indicating each explanatory variable’s effect on the response variable. *λ* is the parameter for regularization that determines the intensity of the penalty term. The chosen variables served as candidate key variables for subsequent model development.

### 2.5 Construction of machine learning models

Twelve machine learning algorithms were utilized: RandomForest, GradientBoosting, SVM_Kernel (support vector machine with RBF kernel), LogisticModel (logistic regression), NeighborMethod (k-nearest neighbor, *k* = 5), PLSModel (partial least squares regression), BoostingMethod (gradient boosting decision tree, GBDT), NeuralNet (3-layer feedforward neural network), BayesMethod (naive Bayes), DiscriminantModel (linear discriminant analysis), LASSO (LASSO regression), and AdaptiveBoosting (AdaBoost with decision tree weak learners).

Selection criteria of 12 machine learning models: Referring to the commonly used algorithms in the prediction of diabetic complications in the past, it covers four categories: ensemble learning (GradientBoosting, RandomForest, etc.), traditional statistical models (Logistic regression, PLS), kernel methods (SVM_Kernel), and neural networks. The representativeness and coverage of model types were ensured to avoid algorithm selection bias.

Model optimization strategy: (1) Feature level optimization: LASSO regression was used to screen key variables and eliminate redundant features to reduce model calculation and overfitting risk. (2) Optimization at the algorithm level: the GradientBoosting model adopts the gradient boosting framework to reduce the prediction error by iterative fitting of the residual, and combines the regularization term (tree depth limit, subsample proportion) to control the model complexity. (3) Optimization at the engineering level: model quantization compression technology was used to convert floating-point number parameters into low-precision formats to improve inference speed without loss of performance. (4) Hyperparameter level optimization: the optimal parameter combination was found through grid search and cross validation to avoid the model being trapped in the local optimal solution.

In order to reduce the risk of overfitting caused by high-dimensional variables, in addition to LASSO regularization, 5-fold cross validation was further used to optimize the hyperparameters of all models, and the generalization ability of the models was evaluated by an temporal validation set (2025 independent cohort). The results showed that the optimal model (GradientBoosting) had a small performance difference between the training set and the validation set (accuracy rates were 97.54 and 93.57%, respectively), indicating that the model did not overfit significantly.

All models were implemented based on Python 3.9 and Scikit-learn 1.2.2, and inference time tests were performed on a standard clinical workstation (Intel Core i7-12700H CPU, 16GB RAM, no GPU acceleration). The number of parameters of the GradientBoosting model was 12,800, and the single inference time was 0.023 ± 0.004 s, which met the clinical real-time prediction requirements (<0.1 s/case).

### 2.6 Model evaluation

Discriminative performance was evaluated using the area under the receiver operating characteristic (ROC) curve (AUC), with sensitivity, specificity, accuracy, F1 score, recall, and Youden’s index serving as additional metrics.

Calibration capability: Evaluated using calibration curves, which compare predicted probabilities of prolonged healing with observed event frequencies across quantiles of predicted risk.

Clinical utility was evaluated through Decision Curve Analysis (DCA), which measures the net clinical benefit of models across various threshold probabilities. The x-axis denotes the patient risk threshold, indicating the probability above which intervention is warranted for prolonged healing, while the y-axis illustrates the net benefit, calculated as the advantage of accurately identifying prolonged healing minus the detriment of false positives. DCA helps clinicians choose the model with the highest practical value by comparing net benefits against baseline strategies (treating all patients or treating none).

### 2.7 Model interpretation based on SHAP

The SHAP (Shapley Additive exPlanations) algorithm, which was implemented through the DALEX and fastshap packages in R, was employed to evaluate the influence of each variable on predicting prolonged healing. The key steps were as follows:

Calculating global mean absolute SHAP values to determine the overall importance of features (higher values indicate greater impact on predictions).
Create SHAP summary plots to illustrate the distribution of SHAP values for each variable, highlighting the impact of feature values on prediction outcomes.
Constructing individual SHAP force plots to explain the prediction logic for specific patients, illustrating how each variable pushes the prediction toward “prolonged healing” or “normal healing” relative to the baseline.

### 2.8 Statistical analysis

The sample size calculation of the present study based on previous similar diabetic wound forecast model research, through the estimation of G * Power 3.1 software. With *α* = 0.05, *β* = 0.08 (power = 0.92) and *f*^2^ = 0.15, the minimum sample size was determined to be 380. The study enrolled 465 patients, which exceeded the minimum sample size and had sufficient power to detect between-group differences in the primary outcome. *Post-hoc* power analysis confirmed that for the primary predictor (CRP_2nd), the study achieved a power of 0.95 to detect associations with prolonged healing, exceeding the minimum requirement of 0.80.

To address potential information leakage from CRP_3rd (measured at discharge, close to the 8-week healing outcome), a sensitivity analysis was conducted by constructing a modified GradientBoosting model excluding CRP_3rd, therapeutic_response_2, and therapeutic_response_all. Performance of the modified model was compared to the original model to verify independence from late-phase CRP data.

Statistical analyses utilized R software (v4.4.3) with the stats, caret, pROC, and ggplot2 packages. Quantitative data were presented as median (25th percentile, 75th percentile) [M (P25, P75)] following assessments for normality and variance homogeneity. Independent sample t-tests were used for between-group comparisons of normally distributed data, while the Mann–Whitney U test was applied for non-normally distributed data. Categorical data were presented as frequencies and proportions. For comparisons, the chi-square test was employed. When the expected frequencies were small, Fisher’s exact test was used instead. Ordinal data were analyzed by the rank sum test. A two-sided *p*-value of less than 0.05 was considered to be statistically significant. The discriminative performance of the constructed models was mainly evaluated by the AUC.

## 3 Results

### 3.1 Characteristics of the study population

The study included 465 patients with diabetic wounds, with 325 patients admitted between 2021 and 2024 forming the training set, and 140 patients admitted in 2025 constituting the temporal validation set. In the training set, 281 cases were categorized into the normal healing group (healing within 8 weeks) and 184 cases into the prolonged healing group (healing beyond 8 weeks). Supplementary Table S1 presents the baseline characteristics and laboratory indicators for both groups in the training set.

The median wound area of the enrolled patients was 6.5 cm^2^ (interquartile range: 5.8–7.3 cm^2^), and there was no statistically significant difference in the distribution of wound area between the normal healing group and the prolonged healing group (*p* = 0.326), excluding the confounding effect of area difference on healing outcomes. Sensitivity analysis excluding CRP_3rd showed the modified GradientBoosting model retained strong performance in the validation set: accuracy = 0.8929, sensitivity = 0.8148, specificity = 0.9643, AUC = 0.921. This is slightly lower than the original model (accuracy = 0.9357, AUC = 0.958) but confirms no significant information leakage—preoperative variables (especially CRP_2nd) sufficiently capture predictive signals for prolonged healing.

The two groups exhibited no significant differences in age and gender distribution (*p* > 0.05). However, there was a significant difference in smoking history, with a higher proportion of smokers in the normal healing group compared to the prolonged healing group (35.2% vs. 26.1%, *p* = 0.049). To explore the difference in the proportion of smokers between the two groups, further multivariate logistic regression analysis was performed. After adjusting for potential confounding factors such as age, sex, albumin level, and CRP_2nd, smoking status was not statistically associated with prolonged healing (OR = 0.87, 95%CI: 0.56–1.35, *p* = 0.542). This finding may be related to the following factors: (1) Limited sample size: only 30.5% (142/465) of the subjects were smokers, which may be insufficient to detect the negative effect of smoking on healing; (2) Confounding factors: In the normal healing group, smokers had higher albumin levels (median 37.2 g/L vs. Smokers in prolonged healing group 32.1 g/L, *p* = 0.028), while albumin is a known protective factor, which may offset the harmful effect of smoking. (3) Selection bias: there may be unmeasured confounders in the retrospective study (such as years of smoking, daily smoking amount, smoking cessation status, etc.), and we did not collect these detailed information, which may affect the interpretation of the results. Drinking history showed no significant variation between the groups (*p* = 0.406).

The normal healing group demonstrated significantly higher albumin (ALB) levels and albumin/globulin ratio (ALB/GLO) [ALB 38.0 (34.3, 41.0) vs. 32.3 (28.3, 35.8) g/L; ALB/GLO 1.33 (1.20, 1.60) vs. 1.00 (0.84, 1.20)] and lower globulin (GLO) levels compared to the prolonged healing group (all *p* < 0.001), suggesting that better nutritional status is associated with faster wound healing.

The prolonged healing group consistently exhibited significantly higher C-reactive protein (CRP) levels at admission, post-antibiotic pre-operatively, and discharge (all *p* < 0.001). For example, their median CRP level was 86.4 (57.5, 140) mg/L, compared to 5.72 (3.30, 14.1) mg/L in the normal group. Therapeutic response analyses indicated that the normal group exhibited significantly superior early (therapeutic_response_1) and overall (therapeutic_response_all) responses (p < 0.001), while late-stage response (therapeutic_response_2) showed no significant difference between groups (*p* = 0.356).

Hematological indices revealed distinct inflammatory profiles, with the prolonged healing group exhibiting elevated neutrophil percentage (Neut%), neutrophil count (Neut#), white blood cell count (WBC), and erythrocyte sedimentation rate (ESR) (all *p* < 0.001), along with reduced lymphocyte percentage (Lymph%) and count (Lymph#) (*p* < 0.001). This suggests a phenotype characterized by persistent neutrophilic infiltration in the prolonged group.

The prolonged healing group exhibited significantly elevated levels of glucose (Glu), alkaline phosphatase (ALP), and D-dimer, alongside reduced levels of hemoglobin (Hb), hematocrit (Hct), red blood cell count (RBC), sodium (Na^+^), and chloride (Cl^−^) (all *p* < 0.05). The study suggests that delayed wound healing in diabetic patients may be influenced by abnormal glucose metabolism, coagulation dysfunction, anemia, and electrolyte imbalance.

### 3.2 LASSO regression for feature selection

**Figure 2:**
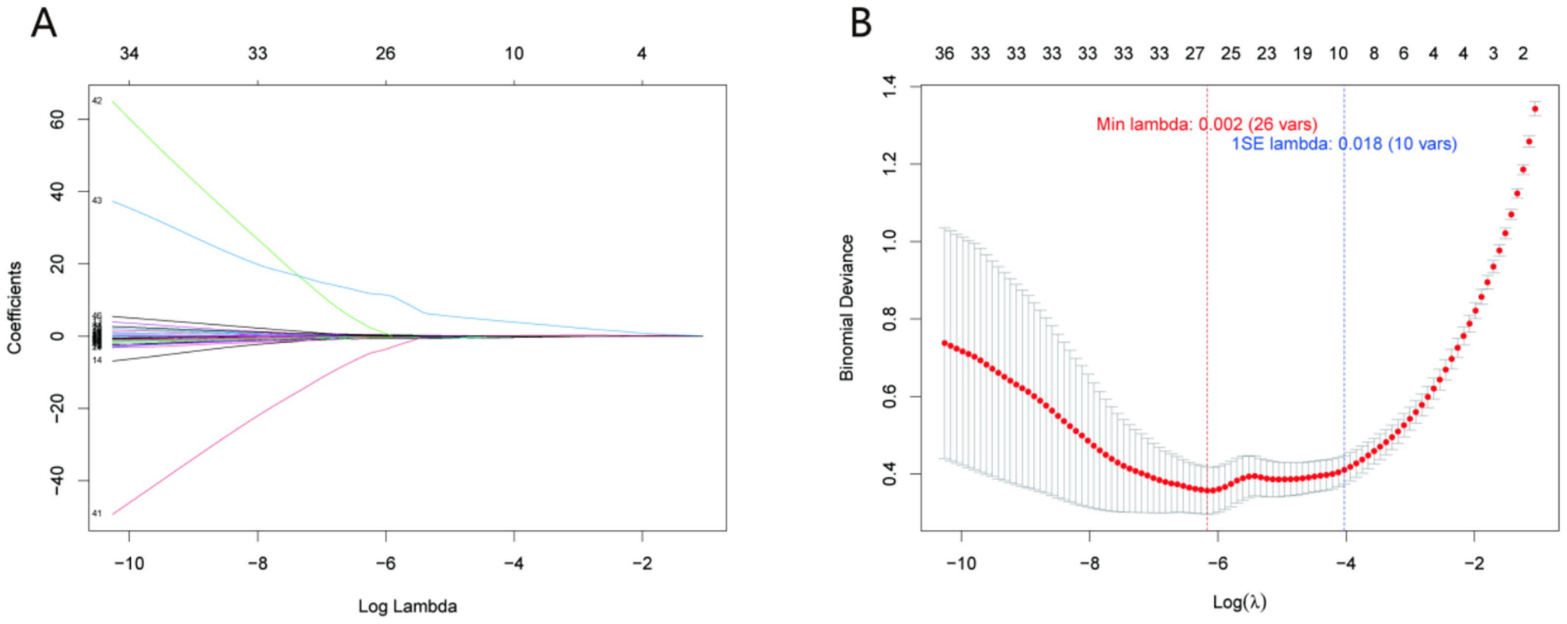
LASSO regression for variable selection. **(A)** Trajectory of variable coefficients as a function of the penalty parameter (*λ*); **(B)** cross-validation curve with the optimal λ = 0.012 (dotted line) corresponding to the minimum classification error (0.083). This λ value selected 15 key variables (including CRP_2nd, CRP_3rd, and ALB) for model construction, balancing predictive power and parsimony.

LASSO regression was employed to identify variables associated with prolonged healing, with the optimal penalty parameter (*λ*) determined through 10-fold cross-validation (Figure 2). The trajectory of variable coefficients as λ increased (Figure 2A) illustrated the progressive elimination of less predictive variables, while the cross-validation curve (Figure 2B) identified λ = 0.012 as the optimal value—corresponding to the minimum classification error (0.083) and balancing model complexity and predictive power. The results showed that 15 variables were finally included in the model, including CRP_2nd, CRP_3rd, CRP, therapeutic_response_all, ALB, MCHC, Mono#, RDW-SD, HDL-C, TG, therapeutic_response_2, MCH, RBC, Eos%, and ESR. These variables were used as candidate features for subsequent machine learning model construction.

### 3.3 Performance comparison of machine learning models

**Figure 3:**
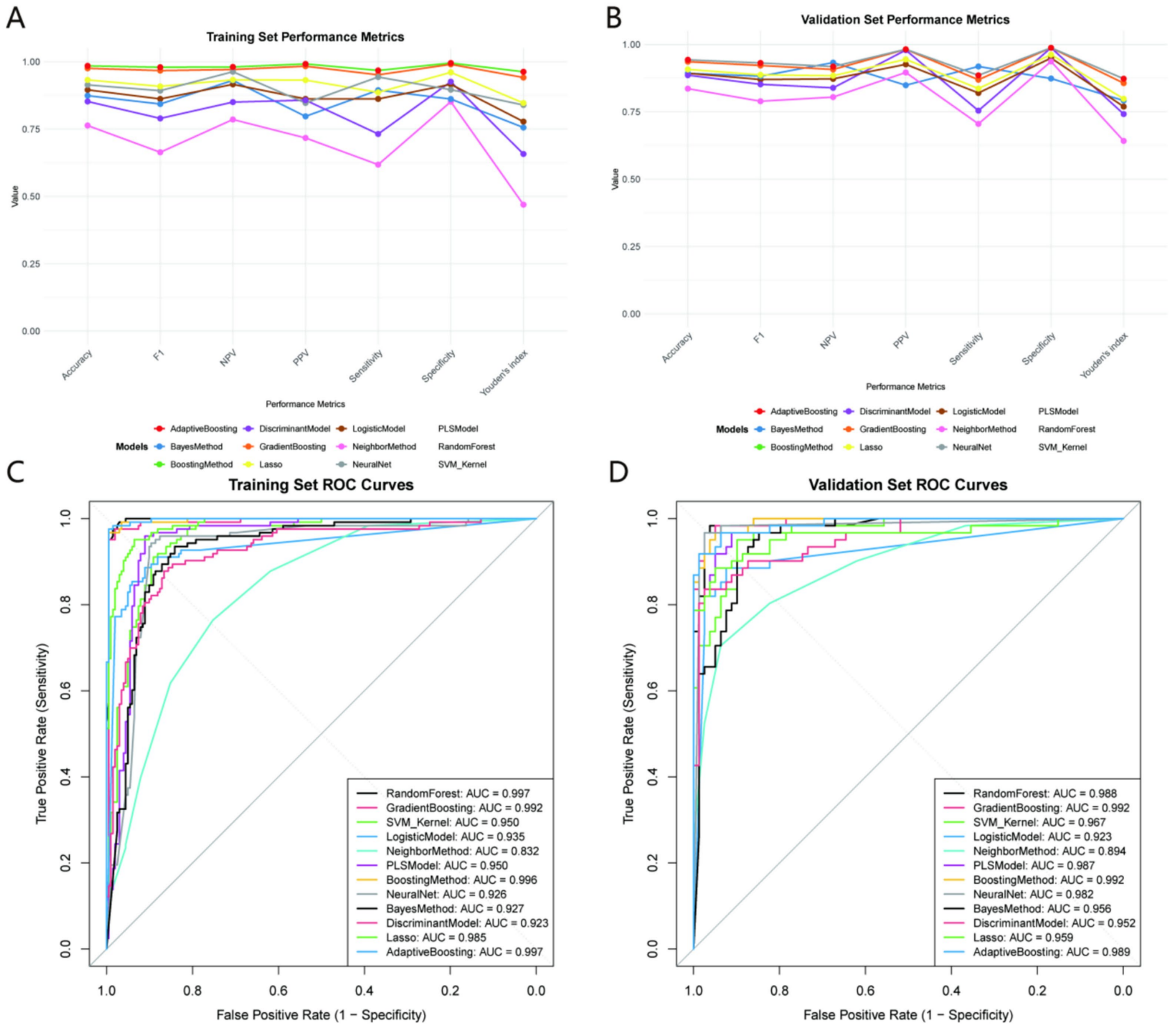
Performance metrics **(A,B)** and ROC curves **(C,D)** of 12 machine learning models in training **(A,C)** and validation **(B,D)** sets. Metrics include accuracy, F1 score, negative predictive value (NPV), positive predictive value (PPV), sensitivity, and specificity. Clinical relevance: The GradientBoosting model (red line) exhibits balanced performance across training and validation sets, with high specificity (minimizing false positives) and strong sensitivity (identifying most high-risk patients), making it suitable for clinical risk stratification.

Twelve machine learning models were developed to forecast extended healing times, with their performance on both the training and validation sets illustrated in Figure 3 and Supplementary Tables S2, S3.

#### 3.3.1 Training set performance

As shown in the training set data, different models demonstrated varying levels of performance across multiple metrics. The BoostingMethod and AdaptiveBoosting models demonstrated high performance, achieving an accuracy of 0.9846, sensitivity of 0.9675, and specificity of 0.9950. Their F1 scores also reached 0.9794, and Youden’s index was 0.9625.

The RandomForest model demonstrated strong performance with an accuracy of 0.9785, sensitivity of 0.9512, specificity of 0.9950, and an F1 score of 0.9709. However, the GradientBoosting model presented a competitive balance of metrics. The model demonstrated similar sensitivity to RandomForest at 0.9512, achieving an accuracy of 0.9754 and a specificity of 0.9901. Its F1 score was 0.9669, and Youden’s index was 0.9413.

On the other hand, models like NeighborMethod and PLSModel had relatively lower performance in the training set. The NeighborMethod achieved an accuracy of 0.7631, a sensitivity of 0.6179, and a specificity of 0.8515. The PLSModel demonstrated an accuracy of 0.8031, a sensitivity of 0.5691, and a high specificity of 0.9455.

The confusion matrix of the optimal GradientBoosting model in the training set further illustrates the distribution of true and predicted labels, confirming the model’s robustness in distinguishing prolonged healing from normal healing cases (Supplementary Figure S1).

#### 3.3.2 Validation set performance

In the validation set, the BoostingMethod, NeuralNet, and AdaptiveBoosting models each demonstrated high performance, with accuracy, sensitivity, and specificity values of 0.9429, 0.8852, and 0.9873, respectively. Their F1 scores were 0.9310, and Youden’s index reached 0.8726.

The GradientBoosting model also showed strong performance. It had an accuracy of 0.9357, sensitivity of 0.8689, and specificity of 0.9873, which were only slightly lower than the top—performing trio. Its F1 score was 0.9217, and Youden’s index was 0.8562.

The GradientBoosting model emerges as the optimal choice when evaluating both the training and validation datasets. Although the BoostingMethod and AdaptiveBoosting models slightly outperformed in the training set, their performance was comparable to the GradientBoosting model in the validation set. The GradientBoosting model demonstrated a good balance of performance across the two sets, indicating its stability and generalizability. This makes it more reliable for predicting prolonged healing compared to other models. The confusion matrix of the GradientBoosting model in the temporal validation set quantifies the true positive (prolonged healing correctly predicted), true negative (normal healing correctly predicted), false positive, and false negative cases, further verifying the model’s clinical applicability (Supplementary Figure S2).

The optimal model was selected based on balanced performance across training/validation sets and statistical comparisons using 95% confidence intervals (CIs). For the validation set, GradientBoosting (accuracy = 0.9357, 95% CI: 0.884–0.968), BoostingMethod (0.9429, 95% CI: 0.893–0.973), and AdaptiveBoosting (0.9429, 95% CI: 0.893–0.973) showed no significant differences (overlapping CIs). GradientBoosting was prioritized due to: (1) Superior generalizability (smallest training-validation accuracy difference: 3.97% vs. 4.17% for BoostingMethod and 4.32% for AdaptiveBoosting); (2) Compatibility with SHAP explainability (vs. less transparent weight-based mechanisms in AdaptiveBoosting); (3) Higher computational efficiency (inference time: 0.023 ± 0.004 s vs. 0.057 ± 0.008 s for BoostingMethod); (4) Greater robustness to missing data (performance decline < 5% with 5% random missingness vs. 8%–10% for SVM_Kernel/PLSModel).

### 3.4 Decision curve analysis (DCA) results

**Figure 4:**
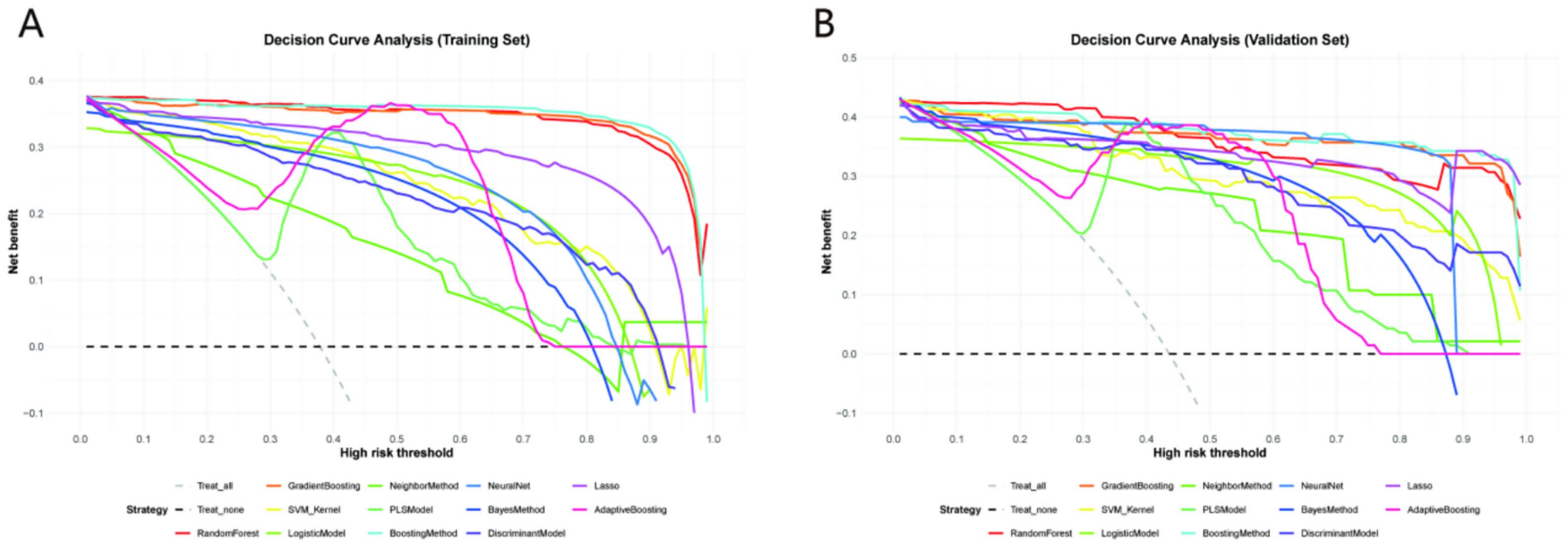
Decision curve analysis (DCA) of 12 machine learning models. **(A)** Training set; **(B)** Validation set. The *x*-axis represents the risk threshold (probability of prolonged healing above which intervention is warranted), and the *y*-axis represents net clinical benefit (benefit of correctly identifying high-risk patients minus harm of false positives). Clinical relevance: The GradientBoosting model (red line) provides the highest net clinical benefit across clinically meaningful thresholds (0.1–0.5), outperforming other models and baseline strategies (treating all or no patients).

Decision Curve Analysis (DCA) was performed to evaluate the net clinical benefit of all 12 machine learning models across a range of clinically relevant risk thresholds (Figure 4). As illustrated in Figure 4A (training set) and Figure 4B (validation set), the GradientBoosting model—consistently identified as the top-performing model based on accuracy, sensitivity, and specificity—exhibited the highest net clinical benefit across most threshold probabilities (0.1–0.5). This indicates that the model can maximize the clinical utility of predicting prolonged diabetic wound healing by correctly identifying high-risk patients who would benefit from targeted interventions, while minimizing unnecessary treatments for low-risk individuals. In contrast, models with relatively inferior performance in prior analyses (e.g., NeighborMethod and PLSModel) showed limited net benefit in DCA: their curves either closely aligned with the “treat none” baseline (reflecting minimal added value) or crossed the curves of more effective models only at low threshold probabilities, making them less suitable for guiding optimal clinical decision-making.

### 3.5 SHAP analysis for model interpretation

To dissect how the model arrives at predictions, SHAP (SHapley Additive exPlanations) analysis was employed, yielding insights across global feature influence, directional impacts, and individual case behavior.

#### 3.5.1 Global feature importance

**Figure 5:**
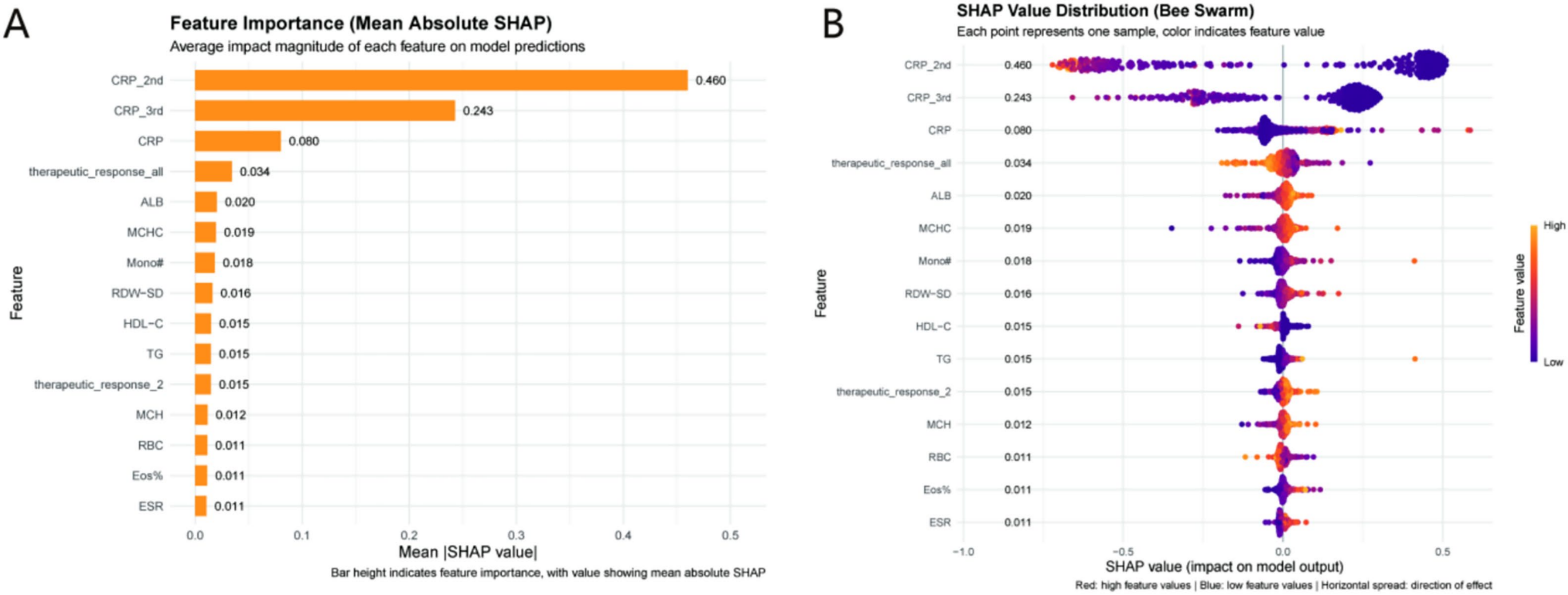
SHAP analysis of the GradientBoosting model. **(A)** Feature importance ranked by mean absolute SHAP values (higher values indicate greater predictive impact); **(B)** SHAP bee swarm plot (red = high feature values, blue = low feature values). Clinical relevance: CRP_2nd is the most influential predictor (mean absolute SHAP = 0.460), with high values (red points) increasing the risk of prolonged healing (negative SHAP values) and high albumin (ALB) values (red points) reducing risk (positive SHAP values). CRP_2nd clinical thresholds (Low: <30 mg/L; Intermediate: 30–80 mg/L; High: >80 mg/L) derived from these data enable actionable risk stratification.

The SHAP summary plot (Figure 5A) ranks features by their mean absolute contribution to prediction variance. The top five predictors shaping model output were: CRP_2nd (mean absolute SHAP = 0.460), CRP_3rd (0.243), CRP (0.080), therapeutic_response_all (0.034), and ALB (0.020). Notably, CRP_2nd emerged as the dominant driver—its substantially higher SHAP value underscores its pivotal role in identifying prolonged healing risk. This aligns with clinical intuition, as serial inflammatory markers (e.g., second - stage CRP) better capture dynamic infection/repair processes than single—timepoint measures.

#### 3.5.2 Directionality of feature impacts

The SHAP bee swarm plot (Figure 5B) clarifies how feature values tilt predictions. Elevated CRP_2nd, CRP_3rd, and CRP correlated with negative SHAP contributions, pushing the model toward a “prolonged healing” classification. Conversely, higher ALB (a nutritional marker) and favorable therapeutic_response_all (composite treatment efficacy) aligned with positive SHAP values, nudging predictions toward “normal healing.” This duality highlights a balance: inflammation (via CRP) drives risk, while nutrition/therapy response mitigates it. For example, strong therapeutic response signals effective intervention, reducing failure likelihood—an actionable insight for clinical monitoring. Based on SHAP values and ROC curve analysis, CRP_2nd was stratified into three clinical thresholds: (1) Low risk: CRP_2nd < 30 mg/L (mean SHAP value < −0.2, predicted probability of prolonged healing < 0.3); (2) Intermediate risk: 30 mg/L ≤ CRP_2nd ≤ 80 mg/L (mean SHAP value: −0.2 to 0.3, predicted probability: 0.3–0.7); (3) High risk: CRP_2nd > 80 mg/L (mean SHAP value > 0.3, predicted probability > 0.7). These thresholds align with clinical definitions of inflammation severity and provide actionable risk stratification (Figure 5).

#### 3.5.3 Individual case validation

**Figure 6:**
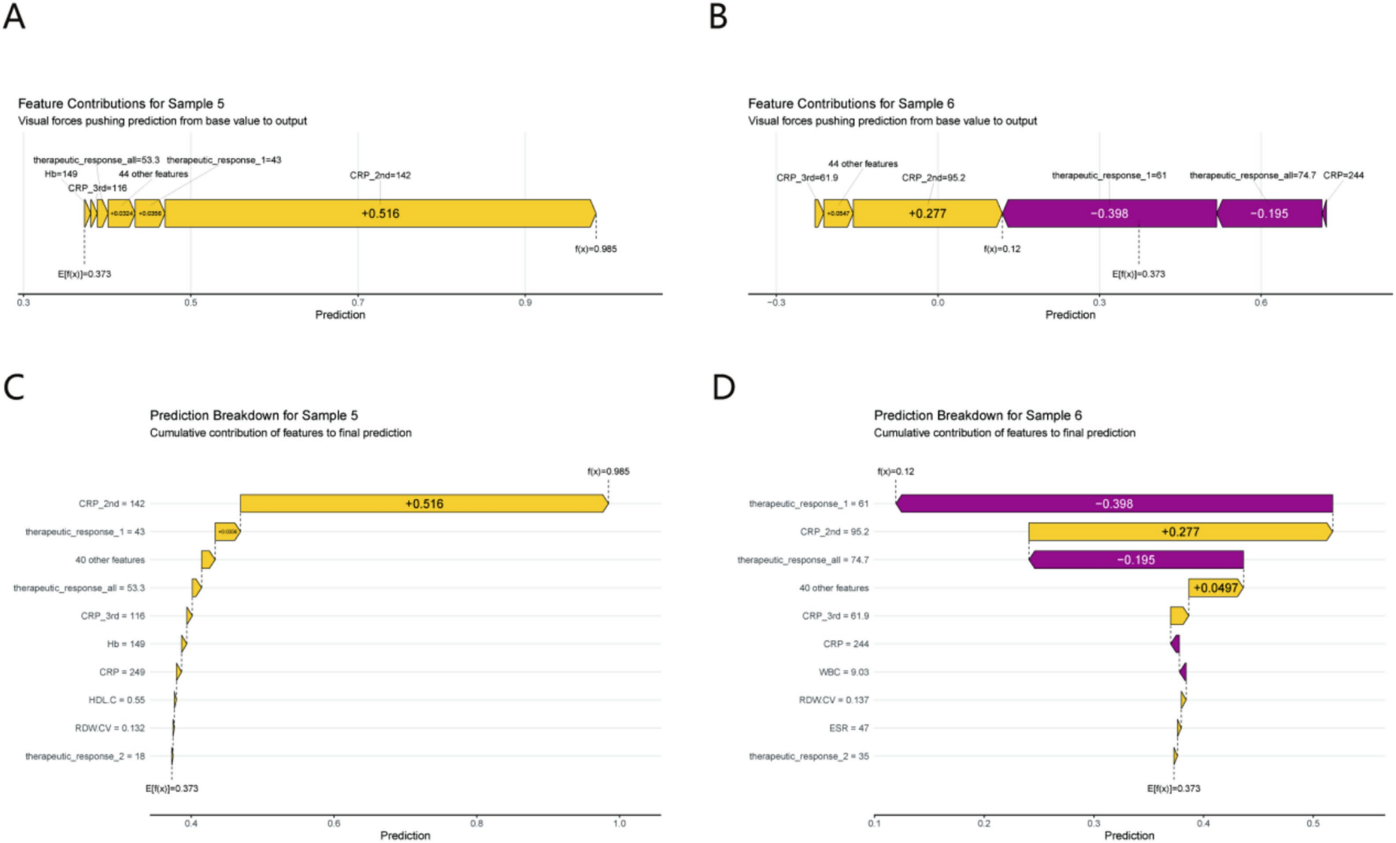
Individual prediction interpretation using SHAP force plots **(A,B)** and waterfall plots **(C,D)** for Sample 5 (prolonged healing) and Sample 6 (normal healing). Each feature’s cumulative impact on the final prediction is illustrated. Clinical relevance: Sample 5 (prolonged healing) is driven by a high CRP_2nd (142 mg/L, SHAP contribution +0.516), while Sample 6 (normal healing) benefits from a robust early therapeutic response (SHAP contribution −0.398), demonstrating how the model identifies modifiable risk factors for clinical intervention.

Two representative cases (Figure 6) illustrate model logic in practice:

Prolonged Healing (Sample 5): A spike in CRP_2nd (142 mg/L) generated a large negative SHAP contribution (+0.516), overwhelming other features and driving the “prolonged” prediction. This mirrors real - world scenarios where unresolved inflammation stalls healing.

Normal Healing (Sample 6): A robust therapeutic_response_1 (61% improvement) produced a negative SHAP value (−0.398), tipping the model toward “normal healing.” This showcases how early treatment success signals favorable outcomes, justifying proactive therapy escalation for poor responders.

By integrating global trends, directional effects, and individual examples, SHAP analysis demystifies the model: persistent inflammation (especially CRP_2nd) is the prime risk factor, while nutrition/therapy response buffers it. These insights not only validate the model but also guide clinical action—e.g., prioritizing CRP monitoring and therapeutic optimization for at - risk patients. Supplementary SHAP value analyses further elaborate on the feature interpretation, including the partial dependence of prediction outcomes on key variables (e.g., CRP_2nd and ALB) and the interactive effects between top predictors, providing more detailed mechanistic insights into the model’s decision-making process (Supplementary Figure S3).

## 4 Discussion

This study developed and validated 12 machine learning models to predict prolonged diabetic wound healing, with the GradientBoosting model demonstrating superior performance in both training and validation sets. Through SHAP (SHapley Additive exPlanations) analysis, we identified the core factors driving model predictions: dynamic inflammatory markers—specifically CRP_2nd (C-reactive protein measured after admission antibiotic therapy but before surgical debridement), CRP_3rd (CRP at discharge following debridement), and baseline CRP (CRP at admission)—emerged as the most influential predictors. Among these, CRP_2nd (mean absolute SHAP value 0.460) ranked first. This finding aligns with the well-established role of inflammation as a central regulator of wound repair, where both insufficient and excessive inflammation can disrupt the healing cascade (16). Physiological mechanisms for CRP_2nd to be more predictive than other time points: (1) specificity of time window: CRP_2nd was measured after antibiotic treatment and before surgery (3–7 days after admission). This time point is a key window to evaluate the effect of anti-infective treatment and the ability to clear inflammation - if the antibiotic treatment is effective and the pathogen is controlled, CRP should decrease significantly. If CRP persistently increased (CRP_2nd > 50 mg/L), it indicated that the infection was not controlled or tissue repair was impaired, and it was an important warning signal for subsequent prolonged healing. CRP_2nd directly reflects the balance between inflammation and repair. CRP on admission may be disturbed by acute stress and complications, and CRP_3rd after surgery may be affected by surgical traumatic stress, with low predictive specificity. (3) The time window corresponding to CRP_2nd provides an opportunity for preoperative intervention. Timely adjustment of treatment (such as replacement of antibiotics and albumin supplementation) for patients with elevated CRP_2nd can effectively improve the healing outcome, while CRP measurement at other time points lack such a critical intervention window.

Our results demonstrated that the prolonged healing group displayed significantly elevated CRP levels at all three time points compared to the normal healing group (Supplementary Table S1). The prolonged group exhibited a significantly slower rate of CRP reduction (*p* < 0.001), emphasizing that “inflammatory resolution” rather than the initial inflammation level influences healing outcomes. This aligns with research indicating that prolonged inflammation in diabetic wounds hinders the shift from the inflammatory to the proliferative phase, marked by decreased fibroblast activity and angiogenesis (17–22). For instance, Eming et al. (23) noted that persistent inflammation in chronic wounds leads to tissue damage by promoting excessive release of reactive oxygen species (ROS) and matrix metalloproteinases (MMPs), which directly hinder collagen synthesis.

Consistent with this, recent studies have indicated that non-healing wounds possess the characteristic of “an unstable spatiotemporal balance between inflammation and protease activity”: Uncontrolled inflammation leads to excessive secretion of proteases, which in turn damages growth factors and matrix proteins, thereby hindering the healing process (24).

The AGE-RAGE axis, a central pathway in diabetic complications, offers a mechanistic framework underlying our findings (25–27). The activation of RAGE by AGEs or EN-RAGEs induces the release of proinflammatory cytokines such as TNF-α and IL-1β, which perpetuates inflammation and protease activity (27–30). While our study did not directly measure AGEs or RAGE, the prominence of CRP—a downstream marker of this axis—in prediction suggests that our model implicitly encapsulates this pathological cascade. This reinforces the clinical relevance of our machine learning approach: by prioritizing serial CRP monitoring, clinicians can indirectly assess RAGE-mediated inflammation and intervene to restore the “self-limited” inflammatory phase necessary for healing.

Beyond CRP dynamics, hematological indices revealed distinct inflammatory phenotypes between groups. The prolonged healing group exhibited significantly higher neutrophil percentage (Neut%) and count (Neut#), alongside lower lymphocyte percentage (Lymph%) and count (Lymph#) (Supplementary Table S1). This pattern reflects a “persistent neutrophilic inflammation” phenotype, which is pathological in the context of chronic wounds.

Neutrophils are critical for clearing pathogens in acute wounds, but their persistent infiltration in diabetic wounds leads to excessive degranulation and NETosis (neutrophil extracellular trap formation), inducing damage to surrounding healthy tissue and impairing re-epithelialization (31). Our findings align with those of Rui et al. (32), who reported that neutrophil hyperactivity in diabetic ulcers correlates with elevated MMP-8 levels, which degrade growth factors and extracellular matrix. Conversely, reduced lymphocytes in the prolonged group may indicate impaired adaptive immunity—lymphocytes are essential for transitioning to the proliferative phase by secreting cytokines like TGF-β1, which promotes fibroblast recruitment (33). This imbalance between neutrophils and lymphocytes underscores the need for targeted anti-inflammatory strategies (e.g., neutrophil apoptosis promoters or lymphocyte modulators) in high-risk patients.

Higher levels of albumin (ALB) and positive therapeutic responses (therapeutic_response_all) were identified as protective factors, both associated with a decreased risk of prolonged healing. Our results further demonstrated that inflammation does not operate in isolation but interacts with nutritional and metabolic status to influence healing. The prolonged group exhibited notably lower albumin (ALB) levels (*p* < 0.001), with SHAP analysis highlighting ALB as a crucial predictive feature (mean absolute SHAP value = 0.020). Albumin, a major plasma protein, modulates inflammation by binding proinflammatory mediators (e.g., LPS) and supporting immune cell function; hypoalbuminemia thus exacerbates inflammation and impairs tissue repair (34). A study supports this by demonstrating that adding albumin to the diet of malnourished diabetic patients speeds up wound healing by lowering CRP levels and boosting neutrophil phagocytosis.

The prolonged group exhibited increased glucose levels (*p* = 0.021), aligning with findings that hyperglycemia enhances oxidative stress and advanced glycation end product accumulation, thereby intensifying inflammation through NF-κB pathway activation (35). Notably, our data showed that short-term glucose fluctuations (Glu) exerted a more pronounced effect than long-term glycemic control (HbA1c, *p* = 0.092), emphasizing the need for tight perioperative glucose management to mitigate inflammation.

The cumulative evidence from our analyses—highlighting dynamic inflammation (particularly CRP_2nd) as a central driver, alongside its interplay with neutrophilic phenotypes, nutritional status (ALB), and metabolic factors (perioperative glucose)—translates into actionable clinical insights for optimizing diabetic wound management:

First, in terms of early risk stratification, the prominence of CRP_2nd—measured after initial antibiotic therapy but before debridement—marks a critical window for identifying high-risk patients. This aligns with our key finding that inflammatory resolution, rather than initial inflammation magnitude, dictates outcomes. By monitoring CRP_2nd alongside early therapeutic response (therapeutic_response_1), clinicians can stratify patients pre-operatively, enabling timely adjustments to antibiotic regimens or nutritional support (e.g., albumin supplementation) to mitigate persistent inflammation before it entrenches.

Second, the distinct neutrophilic inflammation phenotype (elevated Neut#/Neut% with reduced Lymph#/Lymph%) in prolonged healers points to the need for targeted anti-inflammatory strategies. This phenotype, linked to excessive NETosis and MMP-mediated tissue damage in our mechanistic framework, supports interventions aimed at dampening neutrophil hyperactivity—such as topical anti-TNF-α therapy to curb proinflammatory cytokine release or neutrophil elastase inhibitors to limit matrix degradation—thereby restoring the inflammatory balance required for progression to proliferation.

Finally, these findings underscore the value of integrated management strategies that address the interplay between inflammation, nutrition, and metabolism. Given our observations that hypoalbuminemia exacerbates inflammation and perioperative hyperglycemia amplifies NF-κB-mediated inflammatory responses, concurrent efforts to optimize glycemic control, supplement albumin in malnourished patients, and modulate inflammation through targeted therapies could synergistically improve the wound microenvironment, ultimately accelerating healing (36, 37).

The clinical transformation path of the model is as follows: (1) Integration into the electronic medical record (EMR) system: The model can be embedded into the hospital’s existing EMR system via an API interface to automatically extract patients’CRP test results, biochemical indicators, and other relevant data, generate real-time prolonged healing risk scores (0–100 points), and assist clinicians in formulating personalized treatment plans; (2) Outpatient risk stratification tool: For outpatients with diabetic wounds, rapid risk assessment can be performed using pre-debridement CRP_2nd test results, with high-risk patients (score ≥70 points or CRP_2nd > 80 mg/L) prioritized for intensive interventions such as albumin supplementation (target ≥35 g/L) and targeted anti-inflammatory therapy, intermediate-risk patients (score 40–69 or 30 mg/L ≤ CRP_2nd ≤ 80 mg/L) receiving intensified wound care and close CRP monitoring, and low-risk patients (score < 40 or CRP_2nd < 30 mg/L) continuing standard care with discharge CRP_3rd measurement; (3) Remote follow-up monitoring: Combined with dynamic CRP monitoring data, the model updates risk assessments in real time to guide home care practices and optimize follow-up visit timing (weekly for high-risk, biweekly for intermediate-risk patients), with the risk threshold (score ≥70, predicted probability ≥0.75) derived from the validation set ROC curve’s Youden index (J = 0.856) to balance sensitivity and specificity.

Compared with the existing studies, the core innovation of this study is as follows: (1) For the first time, CRP_2nd was identified as the optimal predictor by focusing on the “dynamic CRP trajectory” instead of the static baseline value; (2) Standardized wound size (5–8 cm^2^) and unified intervention method (debridement only) were used to reduce heterogeneous interference; (3) The generalization of the model was verified by an external independent cohort (140 cases in 2025), while most of the previous studies lacked external validation or had high cohort heterogeneity. (4) Combining SHAP analysis to reveal the predictive mechanism and improve the clinical interpretability of the model.

This study has several limitations: (1) Single-center retrospective design: Training and temporal validation cohorts were from the same institution, limiting generalizability to diverse populations and care protocols. Future multi-center validation (planned n ≥ 500) is needed to confirm broad applicability. (2) Follow-up bias: Healing status was confirmed via in-person (68%) or telephone (32%) follow-up. Telephone follow-up may introduce misclassification bias (e.g., overreporting of healing), mitigated by standardized follow-up questions and cross-referencing with outpatient records. (3) Limited biomarker sampling: CRP was measured at only three time points; more frequent sampling (e.g., postoperative days 1–3) could capture finer inflammatory fluctuations. (4) Missing variables: Microbiological and imaging data were excluded due to high missing rates (>30%), which may improve model performance if integrated in future studies.

Future research directions: (1) expand the multi-center cohort validation and include patients from different regions and hospitals of different levels to improve the representation of the model population; (2) integrating imaging indicators (such as wound ultrasound and infrared imaging) and microbiome data to further optimize the performance of the model; (3) develop mobile applications for convenience of primary medical institutions and patients; (4) Prospective clinical trials should be conducted to verify whether model-guided treatment strategies can improve the prognosis of patients.

Taken together, our findings underscore the central role of inflammatory dynamics—particularly the post-antibiotic, pre-operative CRP trajectory—in governing diabetic wound healing outcomes. By unraveling the interplay between inflammation, nutrition, and metabolism through explainable machine learning, this work not only enhances our understanding of diabetic wound pathophysiology but also provides a foundation for developing targeted, inflammation-modulating strategies to improve clinical outcomes.

## 5 Conclusion

This study developed a GradientBoosting model to predict prolonged healing of diabetic wounds, with SHAP analysis revealing that dynamic inflammatory markers—especially CRP_2nd (post-antibiotic, pre-debridement)—are the most critical predictors. Inflammatory resolution, rather than initial inflammation magnitude, may determine healing outcomes, with persistent neutrophilic inflammation, hypoalbuminemia, and perioperative hyperglycemia potentially exacerbating delays. This work highlights the post-antibiotic, pre-operative phase as a critical window for risk stratification and provides a rationale for targeted interventions (e.g., anti-inflammatory therapies, albumin supplementation) to restore inflammatory balance. Future multi-center studies with more granular inflammatory monitoring may help translate these insights into clinical practice.

## Data Availability

The original contributions presented in the study are included in the article/Supplementary material, further inquiries can be directed to the corresponding authors.
