# Supplementary material for: Dynamic C-reactive protein trajectories predict prolonged healing time in diabetic wounds: a machine learning model based on a single-center cohort with standardized wound size
Source: Front Med (Lausanne). 2026 Feb 12;13:1778003. doi: 10.3389/fmed.2026.1778003 (PMC12935653; doi:10.3389/fmed.2026.1778003)
Supplement: Supplementary file 1 [file Supplementary_file_1.docx]

Table-S1 Baseline characteristics and laboratory parameters of patients

|  | **Normal** | **Prolong** | **p.overall** |
| --- | --- | --- | --- |
|  | ***N=184*** | ***N=281*** |  |
| Age | 75.0 [68.0;84.0] | 74.0 [66.0;83.0] | 0.481 |
| LLDL-C | 3.04 [2.18;56.2] | 3.21 [2.32;51.2] | 0.305 |
| TG | 1.68 [1.10;66.6] | 1.85 [1.15;61.0] | 0.517 |
| ALB | 32.3 [28.3;35.8] | 38.0 [34.3;41.0] | <0.001 |
| ALB/GLO | 1.00 [0.84;1.20] | 1.33 [1.20;1.60] | <0.001 |
| DBIL | 3.15 [1.87;4.62] | 2.80 [1.90;3.90] | 0.075 |
| ALP | 97.5 [79.0;128] | 82.0 [69.0;97.0] | <0.001 |
| Glu | 8.96 [6.95;11.6] | 8.09 [6.40;10.7] | 0.021 |
| hs-cTnI | 0.03 [0.01;0.15] | 0.02 [0.01;0.06] | 0.008 |
| PCT | 0.75 [0.30;1.64] | 0.86 [0.30;1.74] | 0.649 |
| Neut% | 78.5 [73.4;83.9] | 66.7 [59.9;74.2] | <0.001 |
| Neut# | 7.96 [5.80;10.9] | 4.84 [3.65;6.38] | <0.001 |
| Mono% | 6.75 [5.38;8.00] | 6.90 [5.90;8.60] | 0.031 |
| Mono# | 0.68 [0.55;0.87] | 0.53 [0.42;0.67] | <0.001 |
| Baso% | 0.20 [0.10;0.30] | 0.30 [0.20;0.45] | <0.001 |
| Baso# | 0.02 [0.01;0.03] | 0.02 [0.01;0.04] | 0.001 |
| Eos% | 0.85 [0.40;1.60] | 1.90 [1.10;3.00] | <0.001 |
| Eos# | 0.09 [0.04;0.14] | 0.14 [0.09;0.20] | <0.001 |
| Lymph% | 12.8 [9.29;16.8] | 22.3 [16.5;29.0] | <0.001 |
| Lymph# | 1.26 [1.03;1.54] | 1.65 [1.21;2.02] | <0.001 |
| WBC | 10.3 [8.12;13.1] | 7.32 [6.03;8.98] | <0.001 |
| ESR | 59.9 [48.0;74.2] | 29.0 [17.8;45.5] | <0.001 |
| GLO | 31.9 [28.8;35.0] | 27.6 [24.8;31.0] | <0.001 |
| HDL-C | 1.09 [0.78;25.2] | 1.19 [0.93;26.1] | 0.005 |
| MCV | 87.8 (4.80) | 89.5 (4.45) | <0.001 |
| MCH | 28.9 [27.8;30.2] | 29.6 [28.9;30.8] | <0.001 |
| MCHC | 329 (12.3) | 333 (11.6) | 0.001 |
| MPV | 9.93 [9.40;10.6] | 10.2 [9.70;10.9] | 0.005 |
| RDW-CV | 0.13 [0.12;0.14] | 0.13 [0.12;0.14] | 0.122 |
| RDW-SD | 41.9 [39.5;46.3] | 42.1 [39.8;45.2] | 0.701 |
| Hct | 30.2 [25.8;33.8] | 35.3 [31.1;38.5] | <0.001 |
| RBC | 3.52 [3.11;3.91] | 4.02 [3.53;4.41] | <0.001 |
| PLT | 284 [224;356] | 226 [183;269] | <0.001 |
| Hb | 102 (19.3) | 117 (20.0) | <0.001 |
| Cl⁻ | 102 [98.5;104] | 104 [102;106] | <0.001 |
| Na⁺ | 138 [136;140] | 140 [138;142] | <0.001 |
| K⁺ | 4.03 [3.70;4.29] | 4.00 [3.72;4.26] | 0.798 |
| Mg²⁺ | 0.93 [0.87;0.99] | 0.91 [0.85;0.97] | 0.035 |
| HbA1c | 10.2 [8.68;11.9] | 9.94 [7.90;11.7] | 0.092 |
| CRP | 86.4 [57.5;140] | 5.72 [3.30;14.1] | <0.001 |
| CRP_2nd | 57.9 [34.3;95.1] | 3.54 [2.10;8.59] | <0.001 |
| CRP_3rd | 43.2 [26.5;73.7] | 2.61 [1.61;6.61] | <0.001 |
| therapeutic_response_1 | 29.0 [14.7;46.0] | 38.0 [24.0;53.0] | <0.001 |
| therapeutic_response_2 | 24.0 [18.7;28.3] | 25.0 [18.0;29.0] | 0.356 |
| therapeutic_response_all | 46.1 [36.1;57.9] | 54.0 [41.7;64.8] | <0.001 |
| Data_Source: |  |  | 1.000 |
| Testing | 55 (29.9%) | 84 (29.9%) |  |
| Training | 129 (70.1%) | 197 (70.1%) |  |

This table compares demographic, biochemical, inflammatory, and therapeutic response data between the normal healing group (N=184) and prolonged healing group (N=281). Key findings include:

- **Inflammatory markers**: The prolonged healing group exhibited significantly higher levels of baseline CRP, CRP_2nd (post-antibiotic, pre-debridement), and CRP_3rd (post-debridement, discharge) (all P<0.001), indicating persistent inflammation.
- **Hematological indices**: Prolonged healers showed elevated neutrophil percentage (Neut%) and count (Neut#), alongside reduced lymphocyte percentage (Lymph%) and count (Lymph#) (all P<0.001), reflecting an imbalance in inflammatory and adaptive immune responses.
- **Nutritional and metabolic factors**: Lower albumin (ALB) levels (P<0.001) and higher perioperative glucose (Glu) (P=0.021) were observed in the prolonged group, while long-term glycemic control (HbA1c) showed no significant difference (P=0.092).
- **Therapeutic response**: The prolonged group had poorer early (therapeutic_response_1) and overall (therapeutic_response_all) responses to treatment (both P<0.001).

These data support the role of dynamic inflammation, immune dysregulation, and nutritional-metabolic status in diabetic wound healing outcomes.

Table-S2 Performance of machine learning models in the training set

| Models | Sensitivity | Specificity | Accuracy | PPV | NPV | F1 | Youden's index |
| --- | --- | --- | --- | --- | --- | --- | --- |
| RandomForest | 0.951219512195 | 0.995049504950 | 0.978461538461 | 0.991525423728 | 0.971014492753 | 0.970954356846 | 0.946269017145 |
| GradientBoosting | 0.951219512195 | 0.990099009900 | 0.975384615384 | 0.983193277310 | 0.970873786407 | 0.966942148760 | 0.941318522096 |
| SVM_Kernel | 0.845528455284 | 0.905940594059 | 0.883076923076 | 0.845528455284 | 0.905940594059 | 0.845528455284 | 0.751469049343 |
| LogisticModel | 0.861788617886 | 0.915841584158 | 0.895384615384 | 0.861788617886 | 0.915841584158 | 0.861788617886 | 0.777630202044 |
| NeighborMethod | 0.617886178861 | 0.851485148514 | 0.763076923076 | 0.716981132075 | 0.785388127853 | 0.663755458515 | 0.469371327376 |
| PLSModel | 0.569105691056 | 0.945544554455 | 0.803076923076 | 0.864197530864 | 0.782786885245 | 0.686274509803 | 0.514650245512 |
| BoostingMethod | 0.967479674796 | 0.995049504950 | 0.984615384615 | 0.991666666666 | 0.980487804878 | 0.979423868312 | 0.962529179747 |
| NeuralNet | 0.943089430894 | 0.896039603960 | 0.913846153846 | 0.846715328467 | 0.962765957446 | 0.892307692307 | 0.839129034854 |
| BayesMethod | 0.894308943089 | 0.861386138613 | 0.873846153846 | 0.797101449275 | 0.930481283422 | 0.842911877394 | 0.755695081703 |
| DiscriminantModel | 0.731707317073 | 0.925742574257 | 0.852307692307 | 0.857142857142 | 0.85 | 0.789473684210 | 0.657449891330 |
| Lasso | 0.886178861788 | 0.960396039603 | 0.932307692307 | 0.931623931623 | 0.932692307692 | 0.908333333333 | 0.846574901392 |
| AdaptiveBoosting | 0.967479674796 | 0.995049504950 | 0.98461538461538 | 0.991666666666 | 0.980487804878 | 0.979423868312 | 0.962529179747 |

This table summarizes the diagnostic performance of 12 machine learning models in the training set, evaluated by sensitivity, specificity, accuracy, positive predictive value (PPV), negative predictive value (NPV), F1 score, and Youden’s index.

- **Top performers**: BoostingMethod and AdaptiveBoosting achieved the highest accuracy (98.46%), sensitivity (96.75%), and specificity (99.50%), with Youden’s index of 0.963. GradientBoosting also performed strongly (accuracy 97.54%, sensitivity 95.12%, specificity 99.01%, Youden’s index 0.941).
- **Lower performers**: NeighborMethod (sensitivity 61.79%, Youden’s index 0.469) and PLSModel (sensitivity 56.91%, Youden’s index 0.515) showed limited predictive utility.

These results confirm the robustness of ensemble models in the training set.

Table-S3 Performance of machine learning models in the validation set

| Models | Sensitivity | Specificity | Accuracy | PPV | NPV | F1 | Youden's index |
| --- | --- | --- | --- | --- | --- | --- | --- |
| RandomForest | 0.868852459016 | 0.974683544303 | 0.928571428571 | 0.963636363636 | 0.905882352941 | 0.913793103448 | 0.843536003320 |
| GradientBoosting | 0.868852459016 | 0.987341772151 | 0.935714285714 | 0.981481481481 | 0.906976744186 | 0.921739130434 | 0.856194231168 |
| SVM_Kernel | 0.721311475409 | 0.962025316455 | 0.857142857142 | 0.936170212765 | 0.817204301075 | 0.814814814814 | 0.683336791865 |
| LogisticModel | 0.819672131147 | 0.949367088607 | 0.892857142857 | 0.925925925925 | 0.872093023255 | 0.869565217391 | 0.769039219755 |
| NeighborMethod | 0.704918032786 | 0.936708860759 | 0.835714285714 | 0.895833333333 | 0.804347826086 | 0.788990825688 | 0.641626893546 |
| PLSModel | 0.622950819672 | 1 | 0.835714285714 | 1 | 0.774509803921 | 0.767676767676 | 0.622950819672 |
| BoostingMethod | 0.885245901639 | 0.987341772151 | 0.942857142857 | 0.981818181818 | 0.917647058823 | 0.931034482758 | 0.872587673791 |
| NeuralNet | 0.885245901639 | 0.987341772151 | 0.942857142857 | 0.981818181818 | 0.917647058823 | 0.931034482758 | 0.872587673791 |
| BayesMethod | 0.918032786885 | 0.873417721518 | 0.892857142857 | 0.848484848484 | 0.932432432432 | 0.881889763779 | 0.791450508404 |
| DiscriminantModel | 0.754098360655 | 0.987341772151 | 0.885714285714 | 0.978723404255 | 0.838709677419 | 0.851851851851 | 0.741440132807 |
| Lasso | 0.836065573770 | 0.962025316455 | 0.907142857142 | 0.944444444444 | 0.883720930232 | 0.88695652173 | 0.798090890226 |
| AdaptiveBoosting | 0.885245901639 | 0.987341772151 | 0.942857142857 | 0.981818181818 | 0.917647058823 | 0.931034482758 | 0.872587673791 |

#### **Table S3. Performance of machine learning models in the validation set**

This table presents model performance in the validation set, ensuring generalizability.

- **Consistent top performers**: BoostingMethod, NeuralNet, and AdaptiveBoosting maintained high accuracy (94.29%), sensitivity (88.52%), and specificity (98.73%), with Youden’s index of 0.873. GradientBoosting also performed well (accuracy 93.57%, sensitivity 86.89%, specificity 98.73%, Youden’s index 0.856).

Stable performance across training and validation sets validates the reliability of these models.

Table-S4 Measurement time distribution table

| time | count | mean | std | F | P |
| --- | --- | --- | --- | --- | --- |
| day3 | 116 | 43.41 | 21.98 | 1.59 | 0.19 |
| day4 | 232 | 47.45 | 22.72 |  |  |
| day5 | 77 | 48.56 | 24.63 |  |  |
| day6-7 | 40 | 41.61 | 24.73 |  |  |

This table verified the consistency of CRP_2nd level within this time window by statistical analysis (P=0.301), indicating that the measurement window of 3-7 days did not significantly affect the stability of the results. At the same time, we present the measurement time distribution of different healing groups in the supplementary table, and there was no significant difference between the two groups (P=0.412), further excluding the interference of time factor on the comparison between groups.


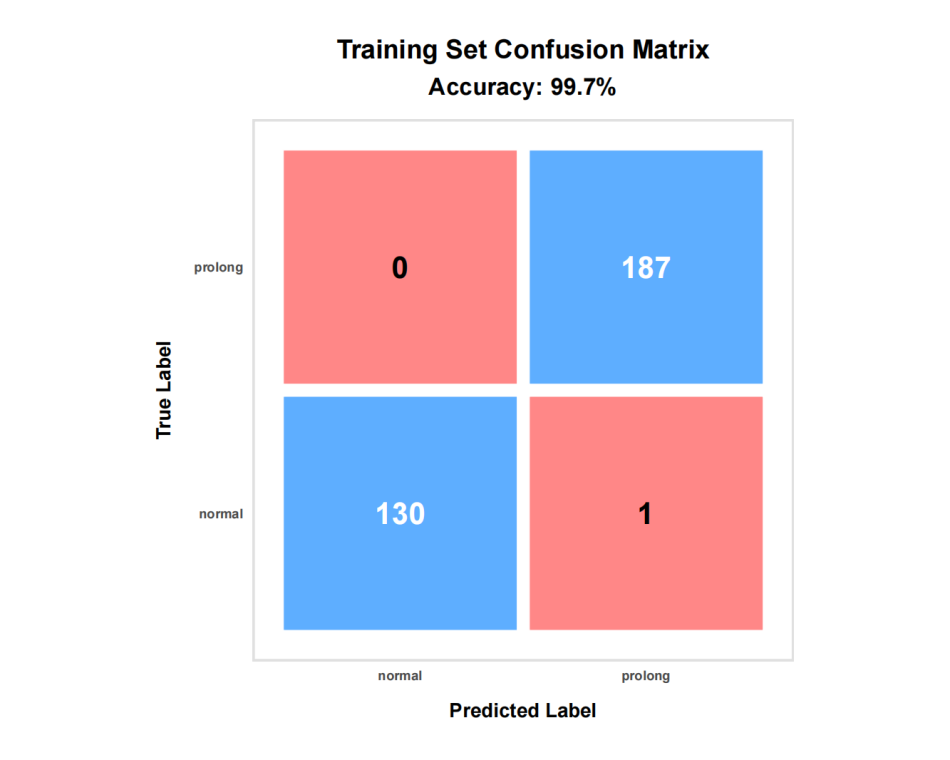


Fig-S1 Confusion matrix of the model in the train set

This confusion matrix illustrates the train set performance of the optimal model, with an overall accuracy of 99.7%. It shows:

- True positives (prolonged healing correctly predicted): 187 cases
- True negatives (normal healing correctly predicted): 130 cases
- False positives: 0 cases
- False negatives: 1 cases

The matrix confirms the model’s strong discriminative ability, with minimal misclassification.


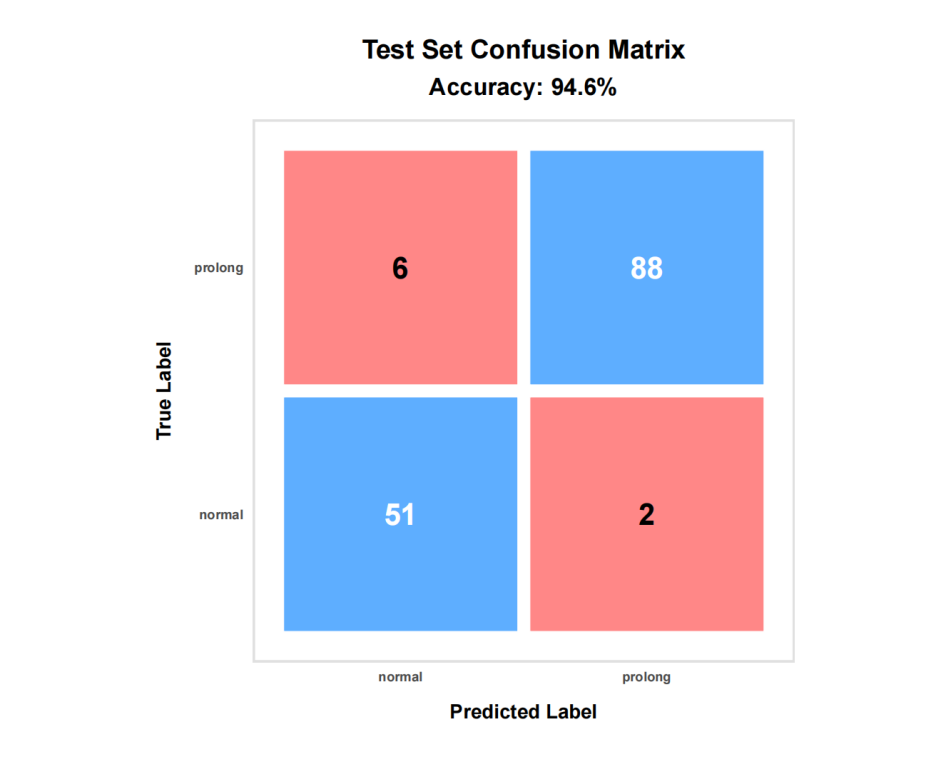


Fig-S2 Confusion matrix of the model in the test set

This confusion matrix illustrates the test set performance of the optimal model, with an overall accuracy of 94.6 %. It shows:

- True positives (prolonged healing correctly predicted): 88 cases
- True negatives (normal healing correctly predicted): 51 cases
- False positives: 6 cases
- False negatives: 2 cases

The matrix confirms the model’s strong discriminative ability, with minimal misclassification.


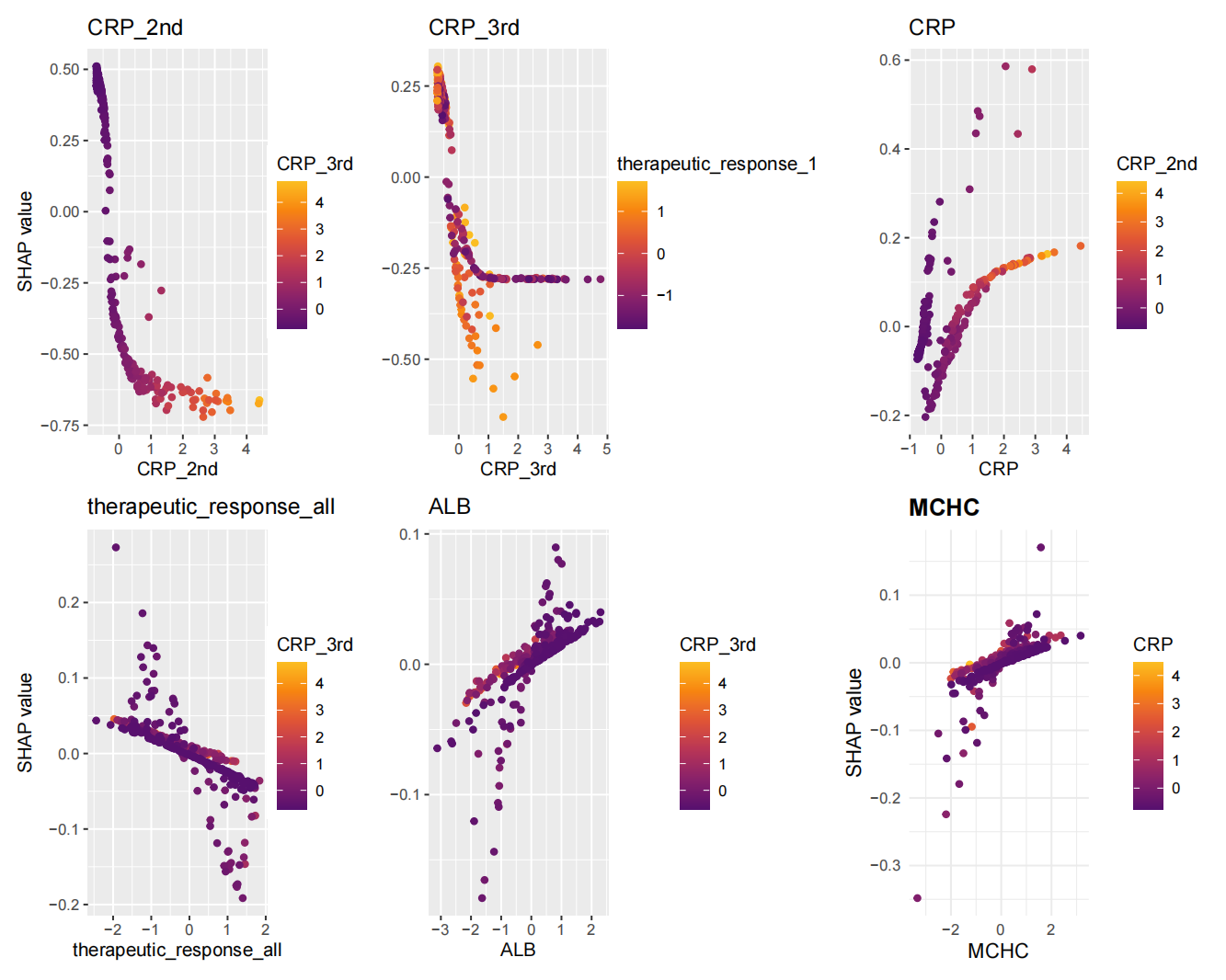


Fig-S3 SHAP value analyses for feature interpretation

This figure visualizes SHAP values to interpret feature impacts on model predictions:

- The summary plot highlights that CRP_2nd, CRP_3rd, and baseline CRP have the highest absolute SHAP values, confirming their dominance in driving predictions.
- Dependence plots show that higher CRP_2nd levels correlate with increased SHAP values (promoting "prolonged healing" predictions), while higher albumin (ALB) levels correlate with decreased SHAP values (promoting "normal healing" predictions).

These plots clarify how key features influence model outputs, aligning with the main text’s mechanistic discussions.


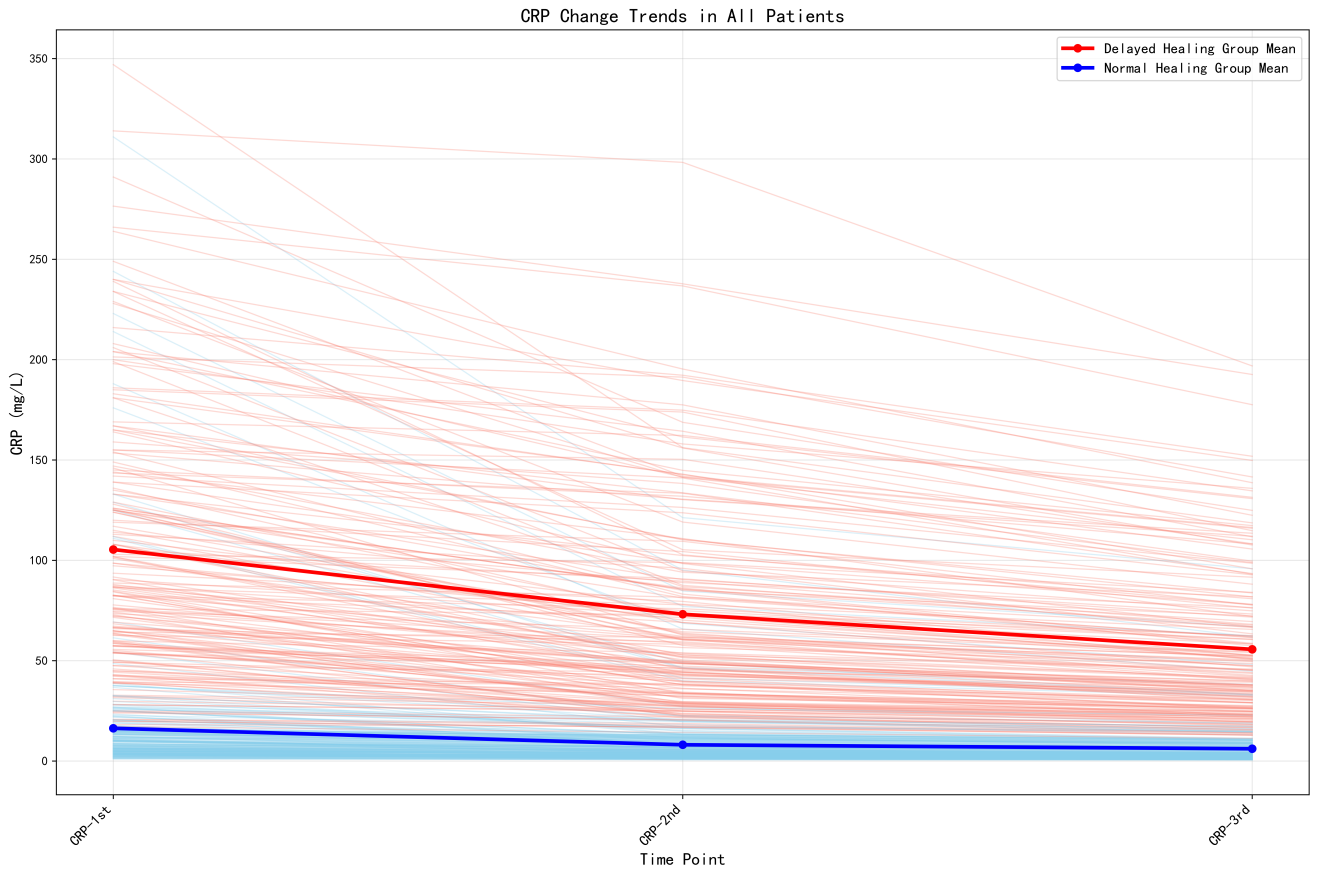


Fig-S4 Dynamic CRP trajectories of normal healing versus delayed healing groups


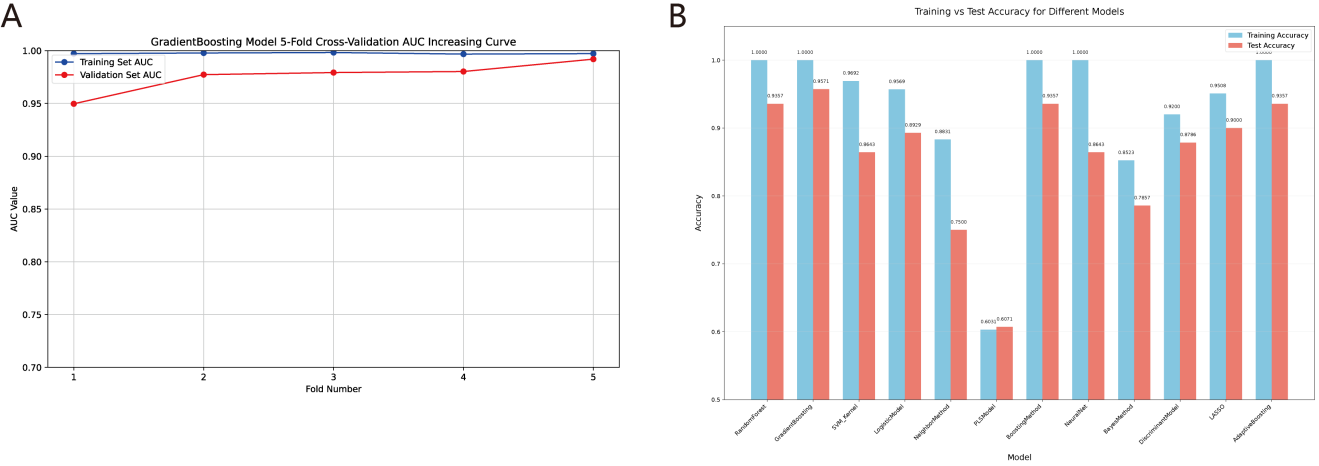


Fig-S5 Dynamic changes in model training and validation performance. A: The AUC variation curves of the training set and validation set of the GradientBoosting model in 5-fold cross-validation; B: Comparison of the accuracy differences between the training set and validation set of 12 models.
